# Nematodes Can Substitute *Artemia* in a Co-Feeding Regime for Pacific White Shrimp Post-Larvae Reared in a Biofloc Nursery System

**DOI:** 10.3390/ani14182679

**Published:** 2024-09-14

**Authors:** Nils Phillip Sommer, Mateus Aranha Martins, Priscila Costa Rezende, Walter Quadros Seiffert, Laurent H. Seychelles, Cláudia Aragão, Felipe Boéchat Vieira

**Affiliations:** 1 Centro de Ciências do Mar (CCMAR/CIMAR LA), Universidade do Algarve (UAlg), Campus de Gambelas, Building 7, 8005-139 Faro, Portugal; nils-sommer@gmx.net; 2 Laboratório de Camarões Marinhos (LCM), Departamento de Aquicultura, Universidade Federal de Santa Catarina (UFSC), Servidão dos Coroas 503, Florianópolis 88061-600, SC, Brazil; m.aranha.martins@gmail.com (M.A.M.); priscila.pesca.ufal@hotmail.com (P.C.R.); walter.seiffert@ufsc.br (W.Q.S.); 3 E-Nema, Klausdorfer Str. 28-36, 24223 Schwentinental, Germany; L.Seychelles@e-nema.de

**Keywords:** live feed, *Penaeus vannamei*, brine shrimp, Nematoda, larval nutrition, BFT

## Abstract

**Simple Summary:**

The use of *Artemia* as a supplement to commercial feed during the nursery period, i.e., from post-larvae 10 up to post-larvae 30, is a common procedure in the shrimp industry. This practice provides larger and more resilient post-larvae (PL) that perform better in grow-out ponds. However, the production of brine shrimp is limited to a few natural salt lakes or man-made salt ponds worldwide, subject to strongly fluctuating yields and product quality. Therefore, ongoing research is focused on finding adequate live food alternatives. Nematodes, commonly known as worms, are animals that greatly fulfil the requirements to be used as live feed for aquatic organisms. Nematodes can be reactivated from their desiccated state by simple rehydration. Additionally, they are rich in protein and fatty acids, making them a favorable nutritional diet for shrimp larvae culture. This study demonstrated that nematodes can substitute *Artemia* in a co-feeding regime for the nursery of shrimp in a biofloc system, increasing the survival of the post-larvae after 20 days.

**Abstract:**

This study evaluated nematodes as an alternative to live *Artemia* when provided in a co-feeding regime to Pacific white shrimp (*Penaeus vannamei*) post-larvae (PL) reared in a biofloc nursery system. A 20-day experiment employing PL_11_ was performed for an evaluation of four dietary groups: control [C] (fed exclusively with a dry commercial feed); artificial *Artemia* [AA] (dry feed composed of extracted and processed *Artemia* cysts and dry commercial feed); live *Artemia* and dry commercial feed [LA]; and nematodes and dry commercial feed [N] (*Panagrolaimus* sp. dry nematodes). The diets were provided twice a day, with the remaining six feed provisions being a commercial dry feed for all experimental groups. A biofloc system was chosen as it offers a potentially sustainable approach to intensive shrimp farming. Alkalinity and pH in the [N] group were significantly higher in the last 5 days of the experiment (*p* < 0.05), likely causing the significantly lower nitrite levels observed in that same group (*p* < 0.05). Total and volatile suspended solids were significantly lower in the two live feed groups when compared with [C] and [AA] (*p* < 0.05). Although PL fed the dry diets exhibited higher growth rates (*p* < 0.05), the [N] and [LA] groups presented significantly higher final survivals (*p* < 0.05). No differences were found for survival after a salinity stress test (*p* ≥ 0.05). These results suggest that nematodes can successfully substitute *Artemia* in a co-feeding regime for *P. vannamei* PL reared in biofloc nursery systems.

## 1. Introduction

In the rearing of the early life stages of Pacific white shrimp (*Penaeus vannamei*), the most cultured crustacean worldwide [1], various live feeds are employed. The use of brine shrimp (*Artemia*) nauplii is a common practice up to post-larvae 10 (PL_10_), after which a dry feed is generally employed as the sole dietary input. However, the use of *Artemia* as a supplement to commercial feeds during the nursery period, i.e., from PL_10_ up to ~PL_30_, is also a general procedure in the shrimp industry [2]. In fact, there is evidence that this live feed supplementation in the nursery phase provides larger and more resilient PL that perform better in the grow-out ponds [3].

Among the reasons for the general use of *Artemia* as a live feed, not only in the shrimp industry but for fish larvae as well, one could cite the possibility of long-term storage of the dry cysts that can be hatched on demand and the possibility of enrichment with essential fatty acids [4,5]. However, since the production of brine shrimp is restricted to a few natural salt lakes or man-made salt ponds around the world that are subject to strongly fluctuating yields and product quality [6,7], there is an ongoing research effort towards finding adequate live food alternatives.

Nematodes, commonly known as roundworms, are animals of the phylum Nematoda that greatly fulfil the requirements to be employed as live feed for aquatic organisms. Their individual size depends on species, but it varies between 150 and 2000 μm in length and 15 and 100 μm in diameter, which puts the smaller individuals in the size class of rotifers and the larger ones in the size class of *Artemia* [8]. In addition to the possibility of being mass produced [9], some species, e.g., *Panagrolaimus* sp. (strain NFS-24-5), can survive a specific dehydration level that makes them eligible for long-term storage, similar to *Artemia* cysts [10]. To reactivate the nematodes from their desiccated state, simple rehydration for one hour is needed, after which they are also able to survive extended periods in saline water. The aforementioned species also synthesizes two essential fatty acids, eicosapentaenoic acid (EPA) and arachidonic acid (ARA), and can be enriched with docosahexaenoic acid (DHA) [11], making it a favorable nutritional diet for shrimp larvae culture. Recently, refs. [12,13] successfully tested *Panagrolaimus* sp. as a live feed for *P. vannamei* during the hatchery phase up to PL_6_ with promising results, although studies for the ensuing phases are lacking. Moreover, in the two cited studies, the shrimp were reared in a system employing daily water exchange, while dietary nematodes have not been assessed for *P. vannamei* when reared in alternative zero-water-exchange systems, such as biofloc technology (BFT), which have been successfully applied to the early phases of this species [14,15]. BFT systems offer a more environmentally friendly approach to intensive aquaculture. For instance, they allow for a successful hatchery phase cycle using approximately 12% of the water used in a conventional autotrophic system [15]. This substantial reduction in water use highlights the importance of evaluating BFT along with novel feeding strategies, especially given the potential bioflocs have of contributing to shrimp nutrition [16].

Considering that the use of nematodes as live feed has not been evaluated for the nursery phase of Pacific white shrimp and the aforementioned evidence for the benefits of live feed supplementation in that rearing stage, this work aimed to evaluate the supplementation of *Panagrolaimus* sp. (strain NFS-24-5) for *P. vannamei* post-larvae reared in a biofloc nursery system as a possible substitute for *Artemia* nauplii.

## 2. Materials and Methods

The experiment was carried out in March 2018 at the Marine Shrimp Laboratory (LCM), part of the Aquaculture Department of the Federal University of Santa Catarina (UFSC), using Pacific white shrimp (*Penaeus vannamei*) post-larvae acquired from Aquatec LTDA (Canguaretama, RN, Brazil). Before stocking in the experimental units, they were maintained in a 4000 L semi-cylindrical tank equipped with proper aeration and heating systems, filled with a mixture of seawater and mature bioflocs, and fed a dry feed (INVE, Nong Lum, Wachirabarami, Phichit, Thailand) according to what is recommended by the manufacturer 8 times a day (8:00, 10:00, 12:00, 14:00, 16:00, 18:00, 21:00, and 24:00) in an acclimation period that lasted four days.

### 2.1. Experimental Design

Four dietary treatments for the nursery of *P. vannamei* post-larvae were evaluated in an experiment lasting 20 days in a completely randomized design in quadruplicate: control [C], fed exclusively with the same commercial feed as used in the acclimation; artificial *Artemia* [AA], dry feed composed of extracted and processed *Artemia* cysts and dry commercial feed; live *Artemia* [LA], live *Artemia* and dry commercial feed; and nematodes [N], *Panagrolaimus* sp. dry nematodes (E-nema GmbH, Schwentinental, Germany) and dry commercial feed. Out of the 8 feed provisions per day (8:00, 10:00, 12:00, 14:00, 16:00, 18:00, 21:00, and 24:00), the special diets were provided at 12:00 and 18:00 (Table 1). For the remaining appointed times, the dry feed was provided for all treatments.

Both the control and the artificial *Artemia* feeds were available in two sizes, which were provided according to what is recommended by the manufacturers (Table 1), whereas the live *Artemia* group received 70 *Artemia* individuals per PL per day, according to [16], which were harvested 24 h after the cysts were incubated. The nematodes were rehydrated 1 h prior to feeding and were provided in a dry weight amount equivalent to that of the live *Artemia*, considering an estimated dry weight for the latter of 1.6 ± 0.1 µg [17] and a 10% addition to compensate for the water still existent in the dehydrated state, yielding 123 µg dry weight of nematodes per PL per day (Table 1). 

The experimental units consisted of 16 semi-cylindrical plastic tanks with 60 L of useful volume. They were equipped with polyvinyl-chloride (PVC) pipes connected to a blower for constant aeration (dissolved oxygen > 6 mg L^−1^), immersion heaters (28 ± 1 °C), and six vertical Needlona ^®^ substrates whose surface area equaled that of the tank, thus doubling the available surface area for the experimental unit. Before stocking, half the volume of the experimental units was filled with filtered seawater (33 g L^−1^), while the other half was filled with mature biofloc water from a shrimp grow-out tank, after which 3600 PL_11_ were stocked in each experimental unit.

The experimental units were managed as biofloc technology units, meaning that no water exchange was performed, with only freshwater being added to replace what was lost through evaporation. Dextrose as an organic carbon source was added to increase the C–N ratio and thus promote the immobilization of ammonia [18], and calcium hydroxide was added to maintain suitable levels of alkalinity, both for the microbial processes [19] and shrimp [20] (Table 2). The added amounts were determined depending on the results from the water analysis for TAN, nitrite, and alkalinity.

### 2.2. Water Quality Analyses

Temperature and dissolved oxygen were measured twice a day with a YSI 55 device (YSI Incorporated, Yellow Springs, OH, USA). Twice a week, total ammonia nitrogen (TAN-N) [21], nitrite (N-NO_2_^−^) [22], alkalinity [23], pH (pHmetro Tecnal^®^), salinity (Eco-Sense YSI EC3), total suspended solids (TSSs) [23], and volatile suspended solids (VSSs) [23] were also measured.

### 2.3. Post-Larvae Performance Assessment

One day before the end of the experiment, a salinity stress test was performed to determine the PL’s ability to survive an osmotic shock. Thirty PL from each experimental unit were randomly selected with a 500 µm sieve and placed in a 500 mL plastic cup containing freshwater at the same temperature as the experimental unit (~29 °C). To minimize handling stress, the whole sieve containing the PL was placed inside the freshwater cup. After 45 min, the sieve was removed and placed in a cup of seawater at 33 g L^−1^ and ~29 °C. A further 45 min later, all surviving and dead PL were counted. PL were determined to be dead when they did not show any movement after stimulus with a plastic probe.

On the final day of the experiment (day 20, PL_31_), the larvae from each tank were collected for the assessment of wet weight (mg), total length (mm), survival (%), and relative growth rate (RGR), the latter being calculated according to the following:

RGR (% day^−1^) = (e^g^ − 1) × 100 (1)

where “e” is the neper number, g = (ln W_t_ − ln W_0_) × t^−1^, W_t_ and W_0_ are the final and initial wet weights, respectively, and t is the duration of the trial in days [24].

### 2.4. Data Analysis

All data were analyzed using the statistical software program R (Version 1.1.453, RStudio, Inc.) [25]. The Shapiro–Wilk test was used to assess the obtained data for normal distribution [26], and Levene’s test determined the homogeneity of variances [27]. When the data were found to be normally distributed and with homogeneous variance, a one-way analysis of variance (ANOVA) [28] followed by Tukey’s honest significant difference (HSD) post hoc test [29] was performed. The water parameters measured over time were analyzed using repeated measures ANOVA, with the treatment group as the main factor and the sampling day as the additional factor. Any data set that was found to be not normally distributed and/or without homogenous variance was analyzed using the non-parametric Kruskal–Wallis rank sum test [30] followed by Dunn’s test [31]. Data are presented as mean ± standard deviation. All significant differences were determined at a confidence level of α = 0.05.

## 3. Results

**Figure 1:**
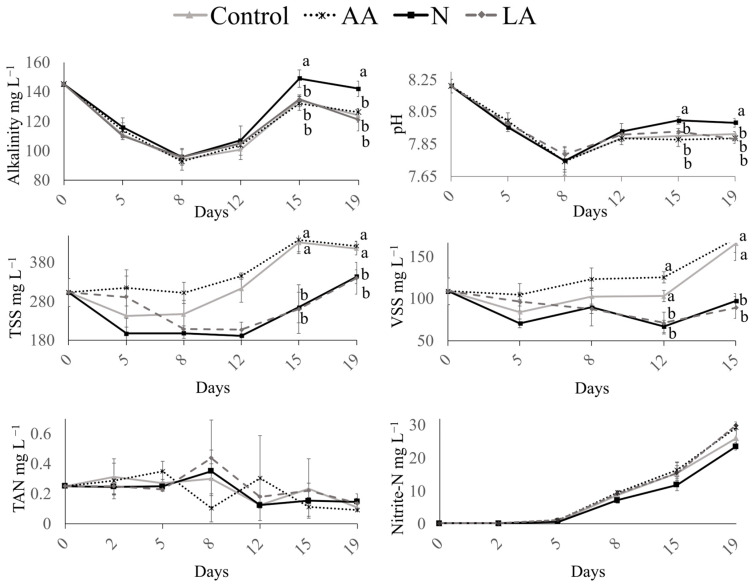
Alkalinity, pH, total suspended solids (TSSs), volatile suspended solids (VSSs), total ammonia nitrogen (TAN), and nitrite concentrations of a biofloc nursery system for the rearing of *Penaeus vannamei* post-larvae fed different dietary treatments (C: control dry feed; AA: artificial *Artemia*; LA: live *Artemia*; and N: nematodes) in a co-feeding regime. Data presented as mean ± standard deviation. Different letters between treatments within any given sampling day indicate statistically significant differences by Tukey’s test (*p* < 0.05). The absence of letters indicates no significant differences (*p* > 0.05).

There was no variation in dissolved oxygen (>7.00 mg L^−1^), salinity (34.48 ± 0.5 g L^−1^), and temperature (28.41 ± 0.31 °C) between treatments. There was a drop in both alkalinity and pH until day 8, which after increased in all treatments. There was no difference between TAN and nitrite levels in the water between all treatments. The ammonia level was lower than 1 mg L^−1^, and there was an increase in nitrite in all treatments during the trial (Figure 1).

A significant distinction between the groups fed with inert diets, [C] and [AA], and live diets, [N] and [LA], started to develop throughout the experiment regarding TSSs and VSSs. On the last two sampling days, day 15 and day 19, the live diet groups had statistically significantly less TSSs than the inert diet groups, indicating a possible effect of the live diet on the biofloc formation itself. At the last sampling on day 15, the groups that received a supplement of live *Artemia* or live nematodes also had statistically significant differences and approximately 50% lower VSS levels than the groups that received only dry feeds (Figure 1).

There was a significant distinction in the final survival of the PL between treatments, with shrimp of the live diet groups exhibiting significantly higher values when compared to the other two. On the other hand, the growth results of wet weight, length, and RGR were statistically significantly higher in the inert diet groups. The stress test results revealed no differences among the groups in survival after exposure to zero salinity (Table 3).

## 4. Discussion

In biofloc systems, the control of toxic nitrogenous compounds is mainly performed by two bacterial processes occurring within the culture water itself, heterotrophic assimilation, the conversion of ammonia directly into bacterial biomass, and nitrification, a two-step process of ammonia oxidation, first into nitrite and then into nitrate [19,32]. Both processes occur simultaneously, and differences between biofloc systems consist of the manager favoring one process over the other, which can be carried out by increasing the C–N ratio through the supplementation of organic carbon to favor heterotrophic assimilation [18] and by using an inoculum containing mature bioflocs to shorten the time for the development of nitrifying bacteria [33,34]. Throughout the experiment, the increasing concentrations of nitrite and the decreasing concentrations of ammonia, the latter being within the safe range for *P. vannamei* [35], suggest that the rates of heterotrophic assimilation and the first oxidation reaction, i.e., ammonia to nitrite by ammonia-oxidizing bacteria (AOB), were adequate for maintaining good water quality as regards ammonia. However, nitrite began to accumulate from day 8 until the end of the experiment, without a clear pattern of reduction. This suggests that the rates of the second step in the nitrification process, i.e., the oxidation of nitrite to nitrate by nitrite-oxidizing bacteria (NOB), were not capable of controlling the buildup of the nitrogenous compound. NOB exhibit a slower growth when compared to AOB, in addition to a higher sensitivity to pH and alkalinity [19,36,37]. Alkalinity dropped continuously and reached the lowest value on the 8th day, when it was below 100 mg L^−1^, the lowest recommended level to support nitrification [19]. pH is positively correlated with alkalinity [38], and its value followed a similar pattern, reaching the lowest value on the 8th day. A reason for the buildup of nitrite occurring in all treatments could be the untimely management of the BFT system in providing a carbonate source and, consequentially, adequate alkalinity and pH values for the nitrifying bacterial community. Instability in the concentration of nitrogenous compounds in biofloc systems is often observed (e.g., [39,40,41]). This could be attributed to it being a highly complex system, where the concentrations of these compounds depend on various interrelated factors, such as bacterial community dynamics, water quality variables, and the physiological processes of the cultured animals [42]. This can lead to fluctuations in nitrification efficiency as the system responds to various internal and external factors.

The highest nitrite concentrations observed were 24–30 mg L^−1^ at day 19. Reference [43] estimated that a concentration of 25.7 mg L^−1^ of nitrite is the upper safe limit level for rearing *P. vannamei* of approximately 4 g at 35 g L^−1^ salinity. Despite the amount of dry feed being reduced by 50% starting on day 16 to reduce the deterioration of the water quality, in the final days of the experiment, the previously mentioned limit was exceeded, meaning that the nitrite accumulation may have had a negative effect on the shrimp’s survival and growth. However, considering that the lower nitrite levels of the [N] group did not result in better growth compared to the other groups, as the highest RGR was observed in the [C] and [AA] groups and survival in the [N] group was also not statistically different from the [LA] one, it can be said that differences between treatments in the larval performances were likely to not have been caused by the accumulation of this compound.

TSS levels between 300 and 600 mg L^−1^ are optimal for rearing *P. vannamei* [44,45]. Within this range, the biofloc nitrogen control is most effective, and the respiration of the shrimp is not handicapped. TSS levels in all groups fluctuated between 200 and 450 mg L^−1^ and were within the desired limits. Hence, a general adverse impact on the shrimp’s growth performance through TSSs is not expected. 

The reasons for the difference in the concentrations of TSSs and VSSs could be threefold. First, throughout the experiment, the live diet groups received approximately 25% less dry feed than the inert diet groups. This difference in inert organic material could have caused divergent developments in the biofloc formation, leading to greater TSS and VSS accumulations in the [C] and [AA] treatments. Furthermore, the feed input was not adjusted to the reduced quantity of PL in the dry feed groups, meaning that it is possible that overfeeding led to an increase in TSSs and VSSs. A second reason could be due to the live feeds themselves. Many free-living nematode species are bacterivores and predominantly feed on bacteria, while some species even occur naturally in biofloc environments [46]. If the *Panagrolaimus* sp. (strain NFS-24-5) survives extended periods of time in salt water, as other nematode species do [6], they may have been ingesting bacteria from the biofloc and thereby reducing the extensive accumulation of VSSs and TSSs in the [N] group. In the case of *Artemia*, they start feeding once they reach the metanauplii stage, which happens after 6 to 8 h at temperatures above 25 °C [47]. Metanauplii are passive filter feeders that will ingest anything small enough, such as microalgae or bacteria [48,49,50]. Since the *Artemia* nauplii were stored for 6 h until the 18h00 feeding at an ambient temperature (~25 °C), it is possible that they had developed into metanauplii by the second special feeding. If nematodes and *Artemia* metanauplii were able to survive predation by the PL, or if they were administered in excess so that a population of live diets remained in the rearing water, they may have consumed the bioflocs and contributed to the significantly lower values of TSSs and VSSs for the live diet groups. Future studies could evaluate this hypothesis by a gut analysis of nematodes and *Artemia* to estimate the rate of their biofloc ingestion and ability to reduce TSSs and VSSs. Finally, the third possibility is that the [N] and [LA] groups received about 25% less dry feed over the course of the experiment, which was supplemented by their specific live diet. If the provided feed was insufficient, it may be possible that the shrimp in these groups, in order to avoid starvation, were grazing more intensely on the biofloc and therefore partially removed it from the system. The final growth results showed that PL from the live diet groups were slightly but still significantly lighter and shorter than the PL from the inert diet groups, which would support the assumption of a suboptimal feeding regime compared to the inert diet groups.

The exact cause of the mortalities and at what moment they occurred remains unclear. The water quality variable, nitrite, whose values could have had deleterious effects on the animals, was comparable among all treatments. Only the [N] group had significantly lower nitrite levels during the final days of the experiment. However, since the [LA] group had a very high survival despite high nitrite levels, it can be suspected that elevated nitrite concentrations were not the sole reason for low survival in the inert diet groups. It may be possible that the shrimp fed with an additional live diet were more capable of surviving in water with lower quality. For instance, while TSS levels were within acceptable ranges, higher values were observed in the two groups with higher mortalities, which may have interacted synergistically with nitrite, negatively impacting shrimp health and survival.

As mentioned above, nematodes and *Artemia* can feed on bacteria from the biofloc [6,49,50] and could thereby bioencapsulate valuable probiotics. Reference [51] suggests adding probiotics in the diet of shrimp to increase their health and resistance to stress and diseases. It is plausible that the live diet groups had increased ingestion of probiotics from the biofloc, which could have improved their survival in elevated nitrite levels compared to the inert diet groups.

Live diets play an important role in the feeding of early shrimp larvae, and complete substitution of live *Artemia* has led to a reduction in survival and overall growth performance in several studies [47,52]. One identified problem that inert diets often pose is the leaching of nutrients to the water, which can impair growth and survival but also degrade water quality [53,54]. Live feeds, such as *Artemia*, are known to be very digestible, and it has been observed that penaeid larvae modulate their enzyme content in the gut in response to dietary quality [55]. Therefore, a live diet supplement may be beneficial for the digestion of inert diets, resulting in better nutrient uptake and increased health of the shrimp. The results of this experiment certainly support this hypothesis regarding health, as final survival in the live diet groups was nearly twice as high as that in the strictly inert diet groups.

The PL from the [C] and [AA] groups were on average 55.5% heavier at the end of the experiment. The length data show the same tendency, that PL from the inert diet groups were significantly different and on average 9.1% longer at the end of the experiment. The RGR also revealed a significant difference between the live and inert diet groups, with the latter growing on average 2.5% more per day. The accumulated findings of the growth parameters point to the conclusion that PL from the two inert diet groups [C] and [AA] were significantly larger at the end of the feeding trial than PL that received a supplement of live diets.

High-protein diets have been shown to enhance the performance in *Penaeus vannamei*. For instance, shrimp post-larvae fed with a diet containing 48.46% crude protein displayed higher survival compared to those fed with lower protein levels [56]. However, excessively high protein levels can lead to metabolic inefficiencies and environmental issues due to increased nitrogen waste, as excess protein is metabolized as an energy source rather than being used for growth, which increases nitrogen excretion into the aquatic environment, reducing water quality and growth performance [57].

The differences in size could be explained by the decreased PL density due to the high mortalities that occurred in the inert diet groups. In addition, the survivors received an excessive amount of feed since the mortalities were unnoticed and the dry feed input was not adjusted. The results in [58] indicate longer larvae length at lower stocking densities during the hatchery phase from mysis to PL_5_. Other studies with older PL also verified that the growth of *P. vannamei* increases with decreasing densities [59,60].

To evaluate the larval quality, salinity stress survival is an important parameter that can ensure that PL will be resistant to transportation and grow-out on a farm. Values above 75% are required by most aquaculture enterprises [61], and all treatments in this experiment had high survival values in the salinity stress test that fulfil this criterion.

Refs. [12,13] have already shown that the nematode species *Panagrolaimus* sp. (NFS 24-5) is capable of replacing *Artemia* nauplii during the early larval stages of *P. vannamei* rearing. Our results provide evidence that this is also the case for the nursery period in BFT, judging from the similar performances between the [N] and [LA] groups under the co-feeding regime. These results are promising, but it is also important to consider economic and logistical factors beyond biological feasibility. *Artemia* cysts, while nutritionally valuable, involve significant costs and infrastructure for hatching and maintenance, requiring controlled environmental conditions and skilled personnel [62,63]. In contrast, nematodes like *Panelograimus* sp. can be cultured more simply and cost effectively, potentially reducing operational costs in aquaculture systems [64]. Additionally, nematodes offer flexibility in production and can be enriched with essential nutrients, making them a viable alternative live feed. The use of nematodes in biofloc technology, which is inherently more expensive due its complex setup and maintenance, could offset these costs by providing a viable alternative to *Artemia* [6].

## 5. Conclusions

Nematodes can successfully replace *Artemia* in a co-feeding regime for *P. vannamei* post-larvae reared in a biofloc nursery system 20-day trial. Shrimp post-larvae fed the nematode co-feeding regime exhibited a similar growth performance and survival to that of the animals fed the live *Artemia* co-feeding regime. There was also no significant difference between survival after a salinity stress test between all four groups (nematode or live *Artemia* co-fed with dry commercial feed, exclusively fed dry commercial feed, and artificial *Artemia* co-fed with commercial dry feed). Overall, the findings support the potential of nematodes as a sustainable live feed substitute in shrimp biofloc technology culture, contributing to more resilient and efficient production systems.

## Figures and Tables

**Table 1:** Feeding schedule used in the evaluation of alternatives to *Artemia* co-feeding for *Penaeus vannamei* post-larvae (PL) reared in a biofloc nursery system.

Day 1–Day 5 (PL_11_–PL_14_) *								
Feeding Times								
Treatment	8:00	10:00	12:00	14:00	16:00	18:00	21:00	24:00
Control [C]	0.28 g DF	0.28 g DF	0.28 g DF	0.28 g DF	0.28 g DF	0.28 g DF	0.28 g DF	0.28 g DF
Artificial *Artemia* [AA]	0.28 g DF	0.28 g DF	0.57 g AA	0.28 g DF	0.28 g DF	0.57 g AA	0.28 g DF	0.28 g DF
Nematodes [N]	0.28 g DF	0.28 g DF	0.22 g N	0.28 g DF	0.28 g DF	0.22 g N	0.28 g DF	0.28 g DF
Live *Artemia* [LA]	0.28 g DF	0.28 g DF	126,000 LA	0.28 g DF	0.28 g DF	126,000 LA	0.28 g DF	0.28 g DF
Day 6–Day 9 (PL_15_–PL_18_) **								
Feeding Times								
Treatment	8:00	10:00	12:00	14:00	16:00	18:00	21:00	24:00
Control [C]	0.36 g DF	0.36 g DF	0.36 g DF	0.36 g DF	0.36 g DF	0.36 g DF	0.36 g DF	0.36 g DF
Artificial *Artemia* [AA]	0.36 g DF	0.36 g DF	0.57 g AA	0.36 g DF	0.36 g DF	0.57 g AA	0.36 g DF	0.36 g DF
Nematodes [N]	0.36 g DF	0.36 g DF	0.22 g N	0.36 g DF	0.36 g DF	0.22 g N	0.36 g DF	0.36 g DF
Live *Artemia* [LA]	0.36 g DF	0.36 g DF	126,000 LA	0.36 g DF	0.36 g DF	126,000 LA	0.36 g DF	0.36 g DF
Day 10–Day 13 (PL_19_–PL_22_) **								
Feeding Times								
Treatment	8:00	10:00	12:00	14:00	16:00	18:00	21:00	24:00
Control [C]	0.45 g DF	0.45 g DF	0.45 g DF	0.45 g DF	0.45 g DF	0.45 g DF	0.45 g DF	0.45 g DF
Artificial *Artemia* [AA]	0.45 g DF	0.45 g DF	0.72 g AA	0.45 g DF	0.45 g DF	0.72 g AA	0.45 g DF	0.45 g DF
Nematodes [N]	0.45 g DF	0.45 g DF	0.28 g N	0.45 g DF	0.45 g DF	0.28 g N	0.45 g DF	0.45 g DF
Live *Artemia* [LA]	0.45 g DF	0.45 g DF	157,500 LA	0.45 g DF	0.45 g DF	157,500 LA	0.45 g DF	0.45 g DF
Day 14–Day 21 (PL_23_–PL_30_) **								
Feeding Times								
Treatment	8:00	10:00	12:00	14:00	16:00	18:00	21:00	24:00
Control [C]	0.56 g DF	0.56 g DF	0.56 g DF	0.56 g DF	0.56 g DF	0.56 g DF	0.56 g DF	0.56 g DF
Artificial *Artemia* [AA]	0.56 g DF	0.56 g DF	0.89 g AA	0.56 g DF	0.56 g DF	0.89 g AA	0.56 g DF	0.56 g DF
Nematodes [N]	0.56 g DF	0.56 g DF	0.35 g N	0.56 g DF	0.56 g DF	0.35 g N	0.56 g DF	0.56 g DF
Live *Artemia* [LA]	0.56 g DF	0.56 g DF	196,875 LA	0.56 g DF	0.56 g DF	196,875 LA	0.56 g DF	0.56 g DF

DF: dry feed. * From day 1 to day 5, DF was given as a 1:1 mix of the sizes “PL” and “XL”, and AA was given as a 1:1 mix of the sizes “Standard” and “Large”. ** Starting from day 6, DF was given only in the feed size “XL”, and AA was given only in the size “Large”.

**Table 2:** Amount of diet, dextrose, and calcium hydroxide added to all groups on each day in an evaluation of alternatives to *Artemia* co-feeding for *Penaeus vannamei* post-larvae (PL) when reared in a biofloc nursery system.

Input	Day *																			
	1	2	3	4	5	6	7	8	9	10	11	12	13	14	15	16	17	18	19	20
Diet (g)	2.2	2.2	2.2	2.2	2.2	2.9	2.9	2.9	2.9	3.6	3.6	3.6	3.6	4.9	4.9	4.9	4.9	4.9	4.9	4.9
Dextrose (g)	-	-	-	-	-	-	-	-	-	-	-	-	-	-	2.5	2.0	1.0	1.0	1.0	1.0
Calcium Hydroxide (g)	-	-	-	-	0.6	0.6	0.6	1.1	1.1	1.1	1.1	0.9	0.9	0.9	0.5	0.5	0.5	0.5	0.5	0.5

* Starting at day 16, the feed was decreased by 50% due to deteriorating water quality.

**Table 3:** Larval performance variables of *Penaeus vannamei* post-larvae fed different dietary treatments in a co-feeding regime when reared in a biofloc nursery system.

Variables	Treatments				
	Control [C]	Artificial *Artemia* [AA]	Live *Artemia* [LA]	Nematodes [N]	*p*-Value
Wet weight (mg)	35.01 ± 10.00 ^a^	35.02 ± 5.78 ^a^	22.50 ± 5.03 ^b^	22.51 ± 5.08 ^b^	0.025
Total length (mm)	17.31 ± 5.02 ^a^	17.13 ± 5.14 ^a^	15.81 ± 3.23 ^b^	15.81 ± 3.95 ^b^	0.002
RGR (% day^−1^)	15.23 ± 1.52 ^a^	15.34 ± 1.02 ^a^	12.83 ± 1.25 ^b^	12.79 ± 1.18 ^b^	0.010
Final survival (%)	51.30 ± 12.08 ^a^	53.16 ± 13.14 ^a^	91.32 ± 8.05 ^b^	93.67 ± 6.97 ^b^	0.001
Salinity stress test survival (%)	90.00 ± 5.08	90.25 ± 8.12	94.25 ± 6.53	87.50 ± 5.79	0.506

Values are means ± standard deviation (n = 4 for all except total length; C: n = 175; AA: n = 189; N: n = 222; LA: n = 246). Different superscript letters within a row indicate significant differences among groups (*p* < 0.05). Initial wet weight was 2 ± 0 mg.

## Data Availability

Data will be made available by the authors upon reasonable request.
